# *Cistanche tubulosa* (Schrenk) Wight Extract Ameliorates Learning and Spatial Memory Abilities in High-Altitude Hypobaric Hypoxia Rats

**DOI:** 10.3390/nu18162744

**Published:** 2026-08-21

**Authors:** Huanhuan Wang, Qiqi Zeng, Weiwen Jing, Xiaojuan Mou, Wenyan Zhao, Dongliang Zhu, Xiaowei Bao, Wenxin Zheng

**Affiliations:** 1 College of Food Science and Pharmacy, Xinjiang Agricultural University, Urumqi 830052, China; 2 College of Chemistry and Chemical Engineering, Xinjiang Agricultural University, Urumqi 830052, China; 3 Xinjiang Key Laboratory for High Value Utilization of Agricultural and Livestock By-Products, Xinjiang Agricultural University, Urumqi 830052, China

**Keywords:** *Cistanche tubulosa* (Schrenk) Wight, high-altitude hypobaric hypoxia, hippocampal, oxidative stress, gut microbiota

## Abstract

**Background:** High-altitude hypobaric hypoxia (HH) is a major environmental stressor that impairs cognitive function, yet effective and widely available therapeutics remain limited. Although *Rhodiola rosea* has shown neuroprotective effects, its resource scarcity restricts large-scale application. *Cistanche tubulosa* (Schrenk) Wight, a traditional Chinese functional food, has been increasingly used in health supplements due to its anti-fatigue, anti-dementia, and memory-enhancing properties, suggesting potential benefits against hypoxia-induced cognitive impairment. **Objective:** This study aimed to investigate the effects of *C. tubulosa* ethanol extract (CTE) on hippocampal tissue and gut microbiota upon chronic HH exposure using SPF male Sprague–Dawley (SD) rats. **Methods:** A total of 60 male SD rats were randomly assigned to six experimental groups (*n* = 10 per group): normoxic control, untreated HH model, positive control (*R. rosea*), and low-, medium-, high-dose CTE treatment groups. Behavioral tests (Morris water maze), hippocampal histopathology, serum and hippocampal oxidative stress markers (SOD, GSH-Px, MDA), expression of PI3K/Akt/mTOR-HIF-1α signaling pathway proteins (by immunohistochemistry), and gut microbiota composition (by 16S rRNA sequencing) were evaluated. **Results:** The results demonstrated that CTE significantly improved cognitive function, enhanced SOD and GSH activities, and reduced MDA levels in both hippocampus and serum. CTE also modulated the expression of PI3K/Akt/mTOR-HIF-1α pathway proteins in the hippocampus. Furthermore, CTE altered gut microbial diversity and abundance, increasing the proportion of beneficial bacteria, which may further influence hippocampal function via the gut–brain axis. **Conclusion:** These findings provide scientific evidence for the application of *C. tubulosa* as a potential health supplement for high-altitude adaptation and lay a foundation for subsequent research.

## 1. Introduction

High-altitude hypobaric hypoxia (HH) is a distinctive environmental stressor that impairs cognitive function through diminished cerebral oxygen supply. Both acute and chronic HH exposure are closely associated with a spectrum of neurocognitive deficits, including impairments in spatial memory, learning, executive function, and sleep quality [1,2,3,4]. The pathophysiology of hypoxia-induced cognitive impairment involves disrupted cellular homeostasis, characterized by dysregulation of redox state, activation of hypoxia-inducible factors, and mitochondrial dysfunction. These stress cascades trigger excessive reactive oxygen species production, leading to oxidative stress, neuroinflammation, and ultimately neuronal apoptosis [5,6].

A wide range of natural products have been reported to protect against HH-induced cognitive impairment, including extracts from *Bacopa monniera* (L.) Wettst. [7], *Withania somnifera* (L.) Dunal [8], *Ganoderma lucidum* (Curtis) P. Karst. [9], *Gynostemma pentaphyllum* (Thunb.) Makino [10], as well as pure compounds such as quercetin [11,12], crocin [13], cordycepin [14], and huperzine A [15]. Based on chemical structures, these agents can be broadly categorized into polysaccharides, flavonoids and other small molecules, and phenylethanoid glycosides. Among these, the phenylethanoid glycosides are of particular interest due to their structural diversity and multi-target neuroprotective activities.

*Rhodiola rosea* L. (*R. rosea*), commonly known as golden root or Arctic root, has been widely used in traditional Chinese medicine to enhance physical and mental resilience against high-altitude stress. Preclinical studies have demonstrated that *R. rosea* extracts and its major phenylethanoid glycoside, salidroside, significantly improve learning and memory in various animal models, including those of HH-induced cognitive impairment [16,17]. Salidroside exerts neuroprotective effects through multiple mechanisms, including antioxidant, anti-inflammatory, anti-apoptotic, and mitochondrial protective activities [18,19,20,21]. Given its well-established efficacy, *R. rosea* serves as an ideal positive control for evaluating new therapeutic candidates against HH-induced cognitive dysfunction. However, the scarcity of *R. rosea* resources limits its large-scale extraction and pharmaceutical application.

Another phenylethanoid glycoside-rich plant, *Cistanche tubulosa* (Schrenk) Wight (*C. tubulosa*), known as “desert ginseng”, has been used in traditional Chinese medicine for tonifying kidney yang and nourishing essence and blood [22,23]. Modern phytochemical investigations have identified phenylethanol glycosides as the principal bioactive constituents of *C. tubulosa*, with echinacoside and acteoside serving as quality markers [23,24]. These compounds share high structural and pharmacological similarity with salidroside, yet the potential of *C. tubulosa* in chronic HH-induced cognitive impairment remains largely unexplored [25,26,27].

A chronic hypobaric hypoxia (HH) rat model was selected to mimic long-term high-altitude environmental stress at 6000 m, which reliably recapitulates the cognitive decline, hippocampal oxidative damage and gut microbial dysbiosis observed in humans exposed to sustained highland hypoxia. Sprague–Dawley (SD) rats were adopted as experimental subjects for the following reasons: First, systematic meta-analyses of hypoxia-cognition preclinical studies demonstrated that SD rats exhibit more stable spatial cognitive readouts and larger intervention effect sizes than mice in Morris water maze tests. Second, sex-specific cognitive divergence exists in rodents; female rats undergo cyclic ovarian hormone fluctuations that introduce unpredictable variability in behavioral and oxidative biomarkers, while male SD rats minimize experimental heterogeneity for spatial memory evaluation. Additionally, SPF-grade SD rats display good tolerance to hypobaric hypoxic exposure and are the most widely utilized strain in high-altitude neuroprotective research [16,28].

To this end, a chronic HH rat model was established using a specialized hypobaric chamber that simulates the hypoxic conditions of Northwest China. Evaluations encompassed spatial memory (Morris water maze), hippocampal histopathology, serum oxidative stress parameters, and immunohistochemical assessment of the PI3K/Akt/mTOR-HIF-1α signaling pathway, alongside 16S rRNA sequencing of gut microbiota. Collectively, this study examines the protective effects of *C. tubulosa* extract (CTE) against chronic HH-induced cognitive dysfunction across behavioral, histopathological, biochemical, and microbiological levels, with *R. rosea* extract as a positive control.

## 2. Materials and Methods

### 2.1. Materials

*C. tubulosa* (Batch No.: 220204) was provided by Hetian Dichen Pharmaceutical Biotechnology Co., Ltd. (Hetian, Xinjiang, China). Hongjingtian capsule (Batch No.: 221004) was provided by Xizang Gaoyuanan Biotechnology Development Co., Ltd. (Lhasa, Xizang, China). Antioxidant indices in the hippocampus were measured using commercial kits (Jiancheng Biotechnology Co., Nanjing, Jiangsu, China), including Superoxide dismutase (SOD), glutathione peroxidase (GSH-Px) and malondialdehyde (MDA). Other reagents were all of analytical grade.

### 2.2. Preparation of CTE

A total of 300 g of dried *C. tubulosa* sample was crushed into 10-mesh coarse powder, and extracted by refluxing with 75% ethanol twice; the solid–liquid ratio was 1:10 (*w*:*v*). The filtrate was combined and then concentrated under reduced pressure to give concentrated extract. And then the extract was treated with macroporous resin AB-8 (200–300 mesh, 1.5 kg), using 65% ethanol to afford the eluate. Finally, the eluate was microwave-dried as the final product.

### 2.3. Determination of the Content of Active Ingredients in CTE

According to the Chinese Pharmacopoeia (2020 Edition, Volume I), the concentrations of echinacoside (Ech-Gs) and acteoside (Act-Gs) were analyzed by the HPLC method [29]. Chromatographic column was TC-C18 column (4.6 mm × 150 mm, 5 μm). The mobile phase consisted of (Phase A) and 0.1% aqueous formic acid solution (Phase B) with gradient elution. The detection wavelength was 330 nm, the flow rate was 1.0 mL/min, the injection volume was 10 μL, and the column oven temperature was set at 30 °C. The chromatographic peak of each analyte was confirmed by matching its retention time with that of the corresponding reference standard. Quantification was performed via peak area integration using the external standard method.

### 2.4. Experimental Animals and Design

#### 2.4.1. Ethics, Husbandry and Animal Source

All experimental procedures were approved by the Animal Ethics Review Committee of Xinjiang Agricultural University (No. 2022004). A total of 80 healthy male SPF Sprague–Dawley (SD) rats, aged 5 weeks and weighing 180–220 g, were purchased from the Animal Experiment Center of Xinjiang Medical University (license No. SYXK (Xin) 2018-0002). Rats were housed at 800 m altitude under controlled conditions: temperature (25 ± 2) °C, relative humidity 40–60%, 12 h light/dark cycle, with free access to standard chow and water. Rats were acclimated for 7 days before experimentation and monitored daily for health and welfare. Predefined humane endpoints for daily monitoring included marked weight loss, decreased food intake and abnormal locomotion to enable timely intervention.

Rodents exhibit inherent sex-specific cognitive biases, and systematic meta-analysis focusing on hypoxic cognitive injury studies further confirmed that single male rats are the mainstream experimental choice for Morris water maze-related spatial memory detection. Mixed-sex cohorts easily introduce unpredictable data heterogeneity. Accordingly, only male SD rats were used to reduce confounding variation; the limitation of this single-sex design is further discussed in the Section 4.

Of the 80 initial rats, 20 rats were used in preliminary pilot experiments to optimize the hypoxia exposure protocol and drug dosages, and were not included in the main study. The 60 rats left that met eligibility criteria were randomly assigned to 6 groups (*n* = 10 per group). No animals were added or removed during the experiment. All 60 rats completed the full experimental protocol without attrition.

This study adopted a randomized, controlled, parallel-group design with repeated measures. A single rat was defined as the independent experimental unit. The six groups included normoxic control, untreated hypobaric hypoxia model, *R. rosea* positive control group, and low/medium/high CTE dose groups. All procedures including MWM spatial memory, hippocampal oxidative stress, hippocampal histopathology and intestinal microbiota were performed to evaluate protective effects of CTE against HH environment.

#### 2.4.2. Inclusion and Exclusion Criteria

All screening standards were established before animal purchase and grouping. Inclusion criteria: healthy 5-week-old male SPF SD rats weighing 180–220 g; normal vision, motor function, and swimming ability; successful acclimation for at least 3 days; and completion of water maze training within 3 min. Exclusion criteria: rats with severe thigmotaxis, persistent floating, drowning, or >3 min latency to locate the platform; rats with illness, injury, or ≥30% missing data. We anticipated that approximately 5–10 rats would be eliminated during pre-grouping behavioral screening according to the above criteria. After screening, zero rats were excluded from the remaining 60 candidate rats reserved for formal experiments, and all eligible animals were allocated to six experimental groups. All screening and elimination procedures were fully completed prior to random group allocation and formal data collection.

#### 2.4.3. Randomization

Rats were randomly assigned to groups using a computer-generated random number list. Allocation was concealed to minimize selection bias. Testing order, cage position, and starting quadrants in the water maze were fully randomized and counterbalanced to control for confounding variables.

#### 2.4.4. Blinding

Investigators performing behavioral testing, video tracking, biochemical assays, immunohistochemistry, and microbiota analysis were blinded to group allocation. Data analysts remained blind to treatment conditions until statistical analysis was completed. Blinded codes were used for all samples and groups to reduce observer bias.

#### 2.4.5. Outcome Measures

Primary outcomes (basis for sample size calculation): MWM spatial learning and memory indexes, measured at pre-hypoxia baseline and 7-day post-hypoxia, including escape latency, central zone residence time and platform crossing frequency in probe trials.

Secondary outcomes included serum and hippocampal SOD, GSH-Px, and MDA levels; hippocampal histomorphology; protein expression by immunohistochemistry; and gut microbial α- and β-diversity.

### 2.5. Animal Experimental Procedures

#### 2.5.1. Hypobaric Hypoxia Model

**Figure 1:**
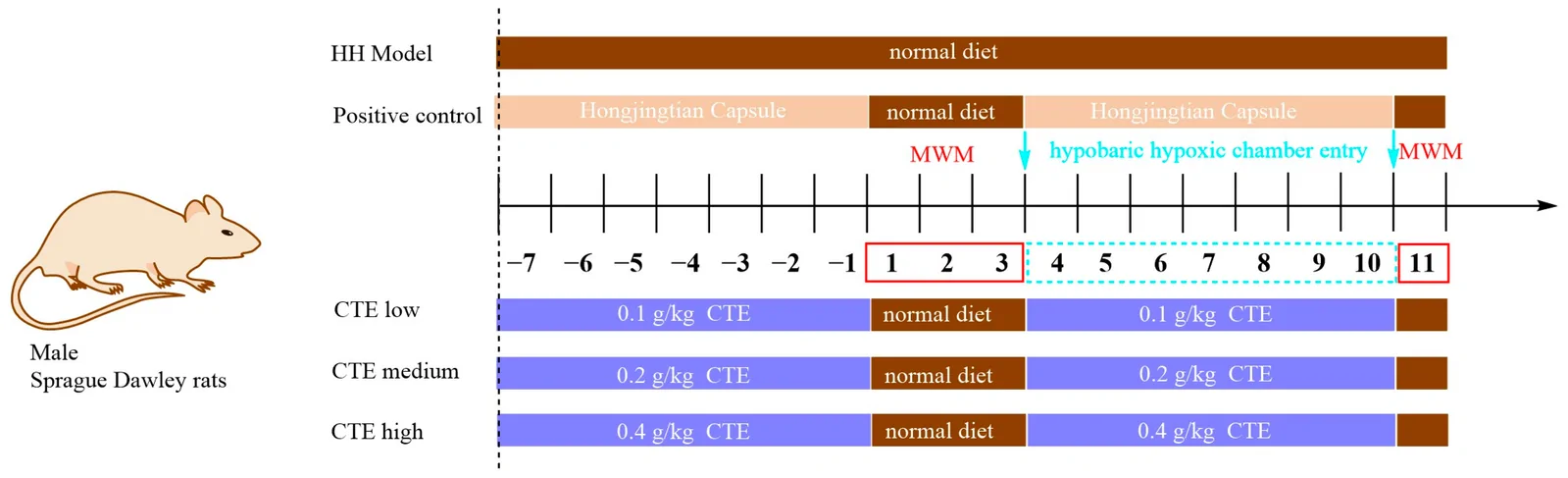
Timeline of adaptive training, training sessions and probe test in the Morris Water Maze (MWM), as well as hypobaric hypoxia (HH) exposure for the CTE groups supplemented with various doses of *C. tubulosa* extract, positive control group, and model group.

As shown in Figure 1, a hypobaric hypoxia model in rats was established by using a hypobaric hypoxia chamber for 7 days, a protocol consistent with established paradigms [8,11]. From days −7–−1, all rats were maintained under normoxic conditions. From days 4–10, all groups except the normoxic control were placed in an artificial hypobaric hypoxic chamber (Feng Lei Oxygen Chamber Co., Ltd., Anshun, Guizhou, China) to simulate an altitude of 6000 m (47.3 kPa). The experimental chamber was set as follows: descending pressure rate of 5.0 m/s, fresh air volume demand of 50 m^3^/h, so that the chamber pressure reaches 47.3 kPa, temperature of 22–24 °C, humidity of 40–50%, and 12 h light-dark cycle. Every morning, the chamber was restored to normal pressure for 1 h to conduct gavage administration, cage cleaning and feed/water replenishment.

#### 2.5.2. Hypoxic Exposure and Drug Administration

The doses used in this study were determined from the recommended dosages for humans and calculated as follows: equivalent experimental dose for rats (mg/kg) = human dose (mg/kg)/body weight (60 kg) × 6.3 [21]. The positive control group was administered *R. rosea* at a dose of 0.24 g/kg. The *R. rosea* was sourced from commercially available Hongjingtian capsules. For CTE intervention, three gradient dosages were set: low dose (0.1 g/kg), medium dose (0.2 g/kg), and high dose (0.4 g/kg), all given by intragastric gavage once daily.

#### 2.5.3. MWM

The maze pool was 1.6 m in diameter, 0.6 m in height, with water depth 0.31 m and temperature (22 ± 2) °C. Fixed distal visual cues were used throughout testing. Rats underwent 3 training trials per day for 3 days. Latency, center activity time, and platform crosses were recorded automatically. Two complete sets of behavioral data were collected for statistical analysis [13].

### 2.6. Rat Hippocampal Tissue and Serum Oxidative Stress Detection

Chloral hydrate was intraperitoneally injected into various groups of rats. After the rats were completely anesthetized, blood was collected from the abdominal aorta and left to stand in a refrigerator at 4 °C for 2 h. The samples were then centrifuged in a refrigerated centrifuge at 3000 rpm for 10 min, and the supernatant was collected. The rats were decapitated and the brain tissues were harvested. The hippocampal tissues were quickly extracted on ice, labeled according to groups, and then stored in a −80 °C freezer. Rat hippocampal tissue, serum SOD and GSH-Px activity, and MDA content measurements were performed according to the manufacturer’s instructions.

### 2.7. Hippocampal Tissue Sectioning and Preparation

Hippocampal tissues were quickly extracted on ice and immersed in 10% formalin solution. Physiological saline was used to wash the tissues four times before dehydration and embedding. Attachment was performed after trimming and sectioning. After xylene clearing for 20 min, hematoxylin and eosin stain was performed and tissue sections were observed.

### 2.8. Immunohistochemistry

Immunohistochemical staining was performed on paraffin-embedded hippocampal sections (4 µm). After deparaffinization with xylene (two changes, 20 min each), sections were rehydrated through a graded ethanol series (100%, 95%, 85%, 75%, 5 min each). Antigen retrieval was carried out by heating sections in 10 mM citrate buffer (pH 6.0) at 95–100 °C for 15 min. Endogenous peroxidase activity was quenched with 3% H_2_O_2_ in methanol for 15 min at room temperature, followed by blocking with 5% normal goat serum (Bioss, Beijing, China) for 30 min at 37 °C. Sections were then incubated overnight at 4 °C with the following primary antibodies: rabbit anti-phospho-AKT (Ser473) (1:100, bs-0871R, Bioss), rabbit anti-phospho-mTOR (Ser2448) (1:500, bs-3498R, Bioss), rabbit anti-phospho-PI3K p85α (Tyr607) (1:100, bs-6417R, Bioss), and rabbit anti-HIF-1α (1:100, bs-0737R, Bioss). After washing with PBS, sections were incubated with HRP-conjugated goat anti-rabbit IgG secondary antibody (1:200, Bioss) for 1 h at 37 °C. Immunoreactivity was visualized using 3,3′-diaminobenzidine (DAB) (ZSGB-BIO, Beijing, China) and counterstained with hematoxylin. Negative controls were performed by substituting the primary antibody with PBS. Finally, sections were dehydrated, cleared, and mounted with neutral resin.

### 2.9. Rat Fecal DNA Extraction, Amplification, and Sequencing

The E.Z.N.A™ Mag-Bind Soil DNA Kit was used to extract rat fecal DNA according to the manufacturer’s instructions. The 16S rRNA V3-V4 hypervariable region-specific primers V3-V4F (5′-CCTACGGGNGGCWGCAG-3′) and V3-V4R (5′-GACTACHVGGGT ATCTAATCC-3′) were used to amplify rat fecal DNA. High-throughput sequencing was employed. The Illumina MiSeq sequencer was used for standardization and sequencing (2 × 300 bp double end) of HH rat feces to obtain raw image data files. After base calling, the data were converted to sequenced reads and the results were stored in the FASTQ format, which includes the sequenced read information and corresponding sequencing quality information.

### 2.10. Data Processing

Sample size was estimated in advance based on preliminary experimental data and previously published hypoxic cognition studies, with significance level α = 0.05 and statistical power set at 0.80. No animals or raw data points were selectively eliminated after random group assignment, and all original experimental records were archived to guarantee data traceability and transparency.

All statistical analyses were conducted with SPSS 26.0, and data were expressed as mean ± standard deviation (x ± s). Intergroup comparisons were performed using one-way ANOVA, followed by post hoc Tukey HSD testing, and *p* < 0.05 indicated statistical significance.

OTU abundance data were normalized to the minimum sample sequencing depth. Alpha diversity reflected intrasample species diversity, and beta diversity analyzed intergroup differences in microbial community structure, which was calculated using QIIME 1.9.1. Iterative methods were applied for PCA and PCoA of sample data.

## 3. Results

### 3.1. The Content of Active Ingredients in the Extract of C. tubulosa Was Determined by HPLC

**Figure 2:**
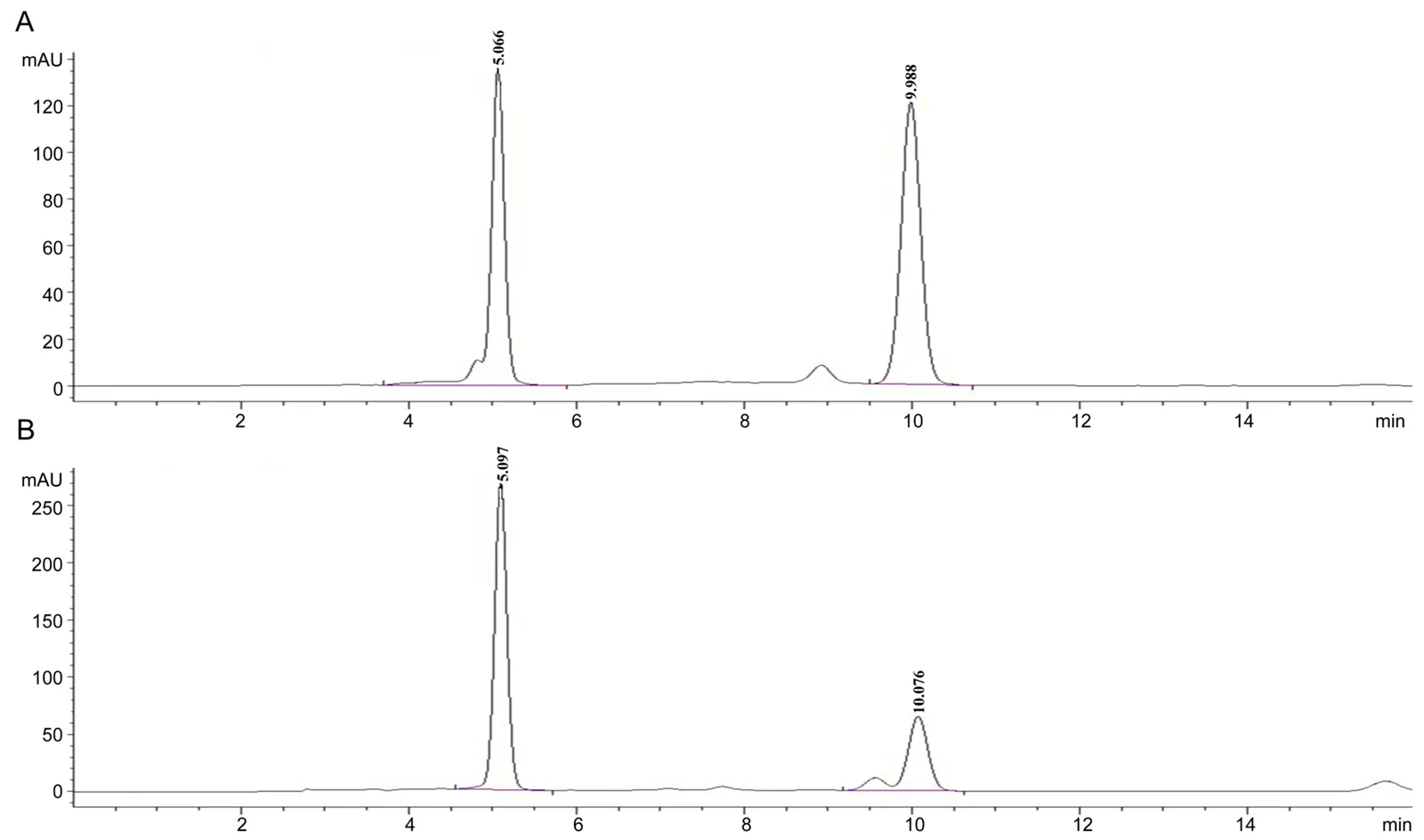
Representative HPLC chromatograms. (**A**) Reference standard chromatogram: peaks 1 (5.066 min) and 2 (9.988 min) correspond to echinacoside (Ech-Gs) and acteoside (Act-Gs), respectively; (**B**) CTE sample chromatogram, with retention times labeled above each peak.

According to the Chinese Pharmacopoeia (2020 Edition, Volume I), echinacoside (Ech-Gs) and acteoside (Act-Gs) are recognized as key marker compounds for the quality control of *C. tubulosa*, the botanical origin of CTE [30]. The quantification of these two active constituents was performed using HPLC. Representative chromatograms of the reference standards and CTE sample are shown in Figure 2. The reference standard chromatogram (Figure 2A) displayed two major peaks at retention times of 5.066 min and 9.988 min, corresponding to echinacoside and acteoside, respectively. The CTE sample chromatogram (Figure 2B) exhibited peaks with identical retention times, confirming the presence of these two active constituents, with retention times labeled above each peak. The standard curve for the production of the standard solution was plotted with the concentration of the standard solution as the *x*-axis and the peak area as the *y*-axis. The linear regression equation for the standard curves of Echinacoside and Verbascoside is Y = 44.505X + 221.1 (R^2^ = 0.999), Y = 19.483X − 9.3831 (R^2^ = 0.9992). Quantitative analysis revealed that the contents of Ech-Gs and Act-Gs in CTE were 236 ± 2.78 mg/g and 12.7 ± 0.57 mg/g, respectively.

### 3.2. MWM Experiment

**Figure 3:**
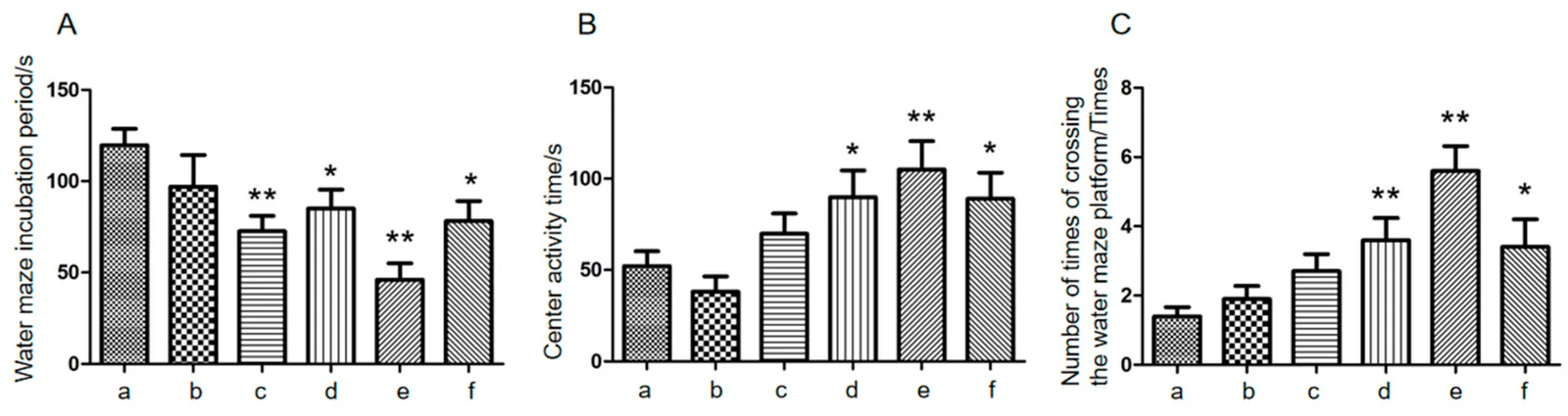
MWM results before entry into the artificial experimental chamber. (**A**): Water maze latency period of different groups of rats. (**B**): Water maze center activity time of different groups of rats. (**C**): Number of water maze platform crosses of different groups of rats. a: Normoxic control group; b: Model group; c: Positive control group; d: Low-dose CTE group; e: Medium-dose CTE group; f: High-dose CTE group. N = 10 rats per group. * *p* < 0.05, ** *p* < 0.01 versus control group.

To evaluate the effects of CTE on cognitive function in rats, the MWM test was performed both before and after hypoxic intervention. Prior to chamber entry, baseline MWM performance was first assessed to ensure intergroup comparability (Figure 3). The results demonstrated that the normoxic control group and the rats subsequently designated for hypoxic modeling exhibited no statistically significant differences in escape latency, center activity time, or platform crossings (*p* > 0.05), confirming equivalent baseline spatial learning, memory, and motor function between the two cohorts before the intervention. In the place navigation trial, compared with the normoxic control group, the low-, medium-, and high-dose CTE groups exhibited decreased escape latencies by 28.9%, 61.5%, and 34.7%, respectively, along with increased center activity times by 72.5%, 101.5%, and 70.8%, and increased platform crossings by 157.1%, 300.0%, and 142.9%, respectively. Statistical analysis revealed that all three CTE doses significantly reduced the latency period and significantly elevated both center activity time and platform crossing number relative to the control group (*p* < 0.05), indicating a dose-dependent improvement in spatial acquisition and memory retention. Notably, the medium dose produced the most pronounced effects across all parameters. This inverted U-shaped dose–response relationship is not uncommon in cognitive enhancement studies. In a dose–response meta-analysis of 58 randomized controlled trials on Omega-3 supplementation, Shahinfar et al. similarly found that the improvement in overall cognitive function followed an inverted U-shaped curve, with efficacy increasing up to an optimal dose and declining thereafter [31]. These converging findings suggest that pharmacological interventions for cognitive improvement often possess an optimal dose window, and that excessive doses may attenuate the beneficial effects, possibly through adaptive negative feedback of the nervous system or non-specific stress responses. Collectively, these findings demonstrate that CTE administration effectively benefit spatial learning and memory in healthy rats.

**Figure 4:**
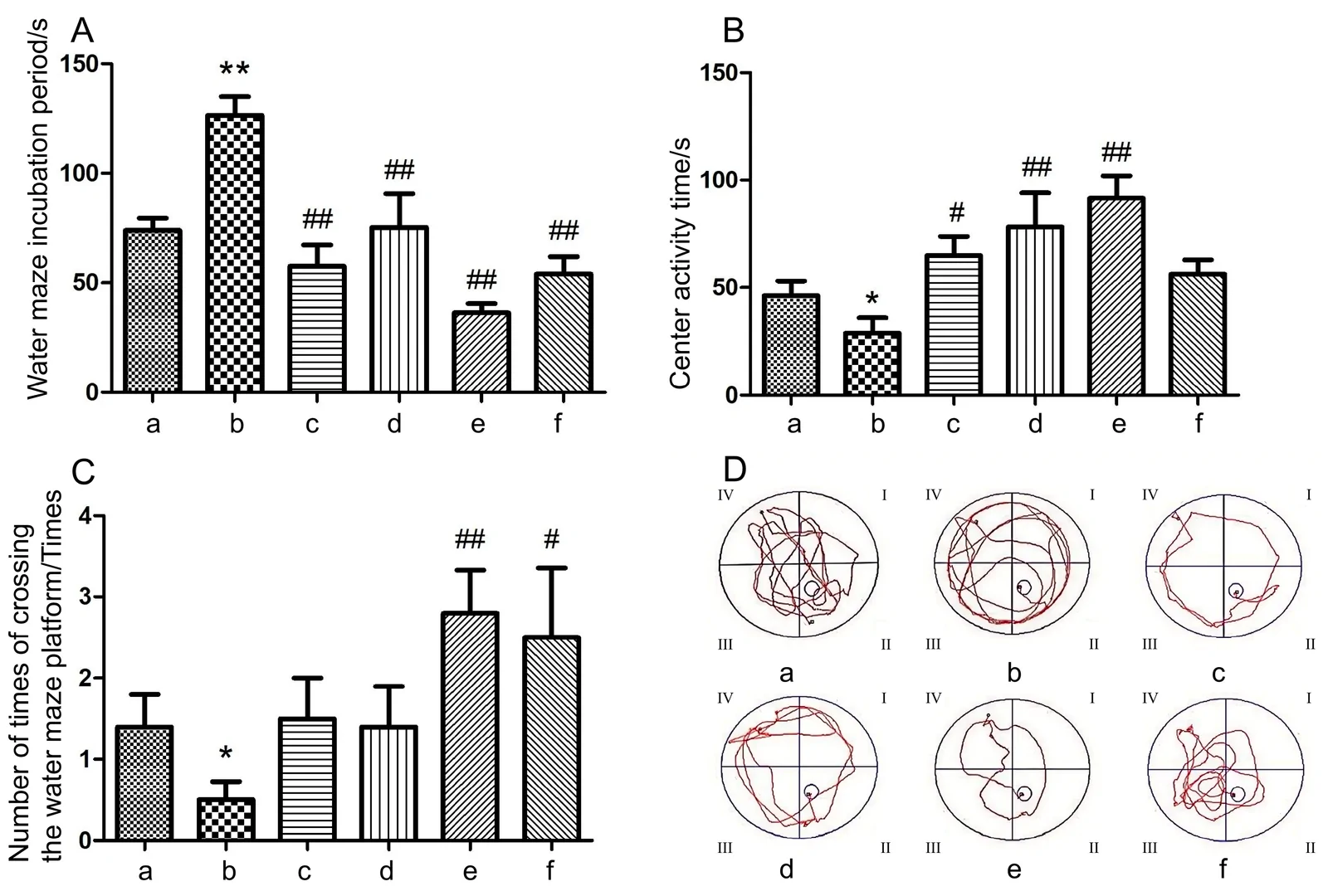
MWM results after entry into the artificial experimental chamber. (**A**): Water maze latency period of different groups of rats. (**B**): Water maze center activity time of different groups of rats. (**C**): Number of water maze platform crosses of different groups of rats. (**D**): Water maze trajectory map of different groups of rats. a: Normoxic control group; b: Model group; c: Positive control group; d: Low-dose CTE group; e: Medium-dose CTE group; f: high-dose CTE group. N = 10 rats per group. * *p* < 0.05, ** *p* < 0.01 versus control group, # *p* < 0.05, ## *p* < 0.01 versus HH group.

MWM test was conducted on day 11, following a 7-day exposure to hypobaric hypoxia (simulated altitude of 6000 m). As shown in Figure 4, the latency period and the center activity time significantly increased while number of platform crosses significantly decreased in comparison between the normoxic control group and model group, indicating that the model construction was successful. Compared to the model group, the latency period of the CTE groups was significantly decreased by 40.4% (low dose), 71.3% (medium dose), and 57.3% (high dose) (*p* < 0.01) (Figure 4A). The center activity time of the low- and medium-dose CTE groups significantly increased by 170.8% and 216.5%, respectively (Figure 4B). The number of platform crosses of the medium- and high-dose CTE groups significantly increased by 460.0% and 400.0%, respectively (*p* < 0.01) (Figure 4C). Hence, it can be seen that 7 days of a HH environment resulted in a memory decline in rats and that CTE could somewhat alleviate memory decline caused by hypobaric hypoxia, of which the medium-dose group had the most significant results. Compared to the normoxic control group, the model group showed a significant edge search strategy, showing that hypobaric hypoxia indeed led to cognitive impairment in rats. Compared to the model group, the various treatment groups showed significantly shortened platform search routes and trajectories, indicating that cognitive impairment was alleviated after CTE was administered to rats, of which the medium-dose group had the most significant results (Figure 4D). As a classic adaptogen for high-altitude conditions, the Hongjingtian capsule produced certain improvements in escape latency and center activity time in this experiment, but failed to demonstrate a statistically significant effect on platform crossings (*p* > 0.05). This dissociation implies that *R. rosea* mainly relieves hypoxic hypoactivity and improves simple spatial preference, with limited effects on spatial memory. In comparison, medium and high doses of CTE raised platform crossings, indicating CTE exerts obvious protective effects on hypoxia-impaired spatial cognition. Such distinction highlights the superior memory-protective efficacy of CTE relative to *R. rosea*, with the hippocampus as a critical target region.

### 3.3. SOD and GSH-Px Activity and MDA Content Measurements

From Table 1, it can be seen that compared to the normoxic control group, the hippocampal tissue SOD activity was decreased by 26.06% in the model group (*p* < 0.01); the SOD activity in the medium- and high-dose CTE groups was increased by 20.98% and 23.90%, respectively (*p* < 0.01); and there was no significant difference in the low-dose group. Compared to the normoxic control group, the serum SOD activity decreased by 19.74% in the model group (*p* < 0.01); the SOD activity in the low- and medium-dose CTE groups increased by 7.39% and 9.04%, respectively (*p* < 0.01); and there was no significant difference in the high-dose group.

Table 2 indicates that compared to the normoxic control group, the GSH-Px activity hippocampal tissue was decreased by 35.17% in the model group (*p* < 0.01). Compared to the model group, the GSH-Px activity was increased by 21.87%, 36.31%, and 24.02% in the low-, medium-, and high-dose CTE groups, respectively (*p* < 0.01). Compared to the normoxic control group, the serum GSH-Px activity was decreased by 28.93% in the model group (*p* < 0.01). Compared to the model group, the GSH-Px activity was increased by 25.84% and 26.22% in the medium- and high-dose CTE groups, respectively (*p* < 0.01), while there was no significant difference in the low-dose group.

Table 3 demonstrates that compared to the normoxic control group, the hippocampal tissue MDA level increased by 61.82% in the model group (*p* < 0.01). Compared to the model group, the MDA levels decreased by 17.61%, 42.25%, and 34.51% in the low, medium, and high-dose CTE groups, respectively (*p* < 0.05). Compared to the normoxic control group, the serum MDA levels were increased by 54.83% in the model group (*p* < 0.01). Compared to the model group, the serum MDA levels were decreased by 16.78% and 21.67% in the low- and medium-dose CTE groups, respectively (*p* < 0.01), while there was no significant difference in the high-dose group.

Exposure to an HH environment induces stress and injurious changes, leading to significantly decreased SOD activity and elevated lipid peroxidation levels [32]. Our study observed a non-monotonic dose–response relationship for CTE, in which the moderate dose demonstrated optimal efficacy in restoring SOD and GSH activities and reducing MDA levels, whereas the high dose showed diminished protective effects. The reduced efficacy or even detrimental effects of higher CTE doses may be attributed to GSH depletion and redox imbalance. This phenomenon likely results from the pro-oxidant potential of excessive phenylethanol glycosides at supra-optimal concentrations, which could overwhelm the endogenous antioxidant defense system or induce metabolic saturation.

### 3.4. CTE Improves Hippocampal Tissue Injury

**Figure 5:**
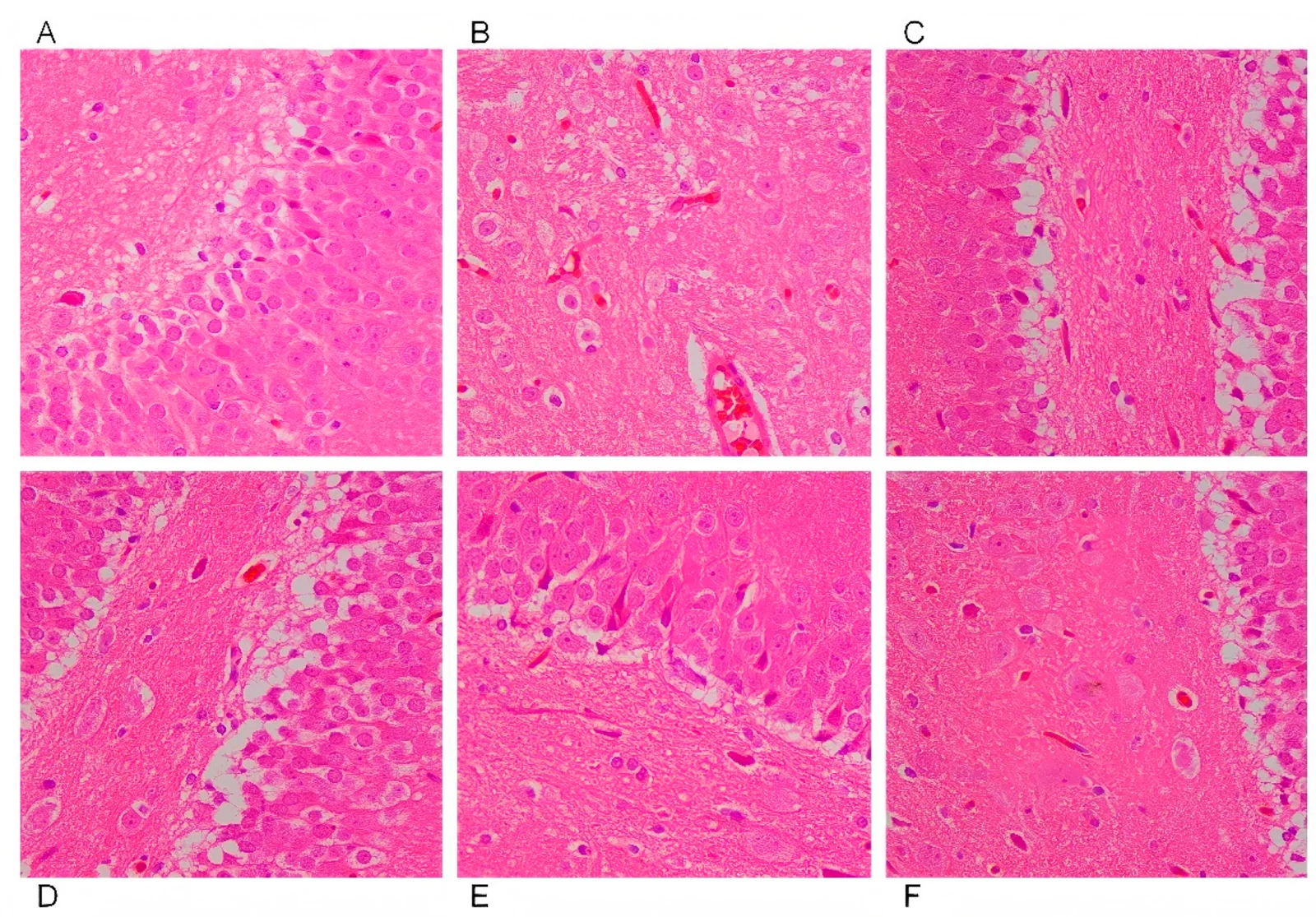
Rat hippocampal tissue section HE staining results (400×). (**A**): Normoxic control group; (**B**): model group; (**C**): positive control group; (**D**): low-dose CTE group; (**E**): medium-dose CTE group; (**F**): high-dose CTE group.

To further study the effector mechanisms of CTE in improving cognitive impairment in rats, we performed HE staining of hippocampal tissues. In the normoxic control group, rat hippocampal tissue showed an intact structure, neat and orderly arrangement, no abnormality in the neurons, and no congestion (Figure 5A). In the hypoxic model group, rat hippocampal tissues showed disorderly structure, with some neurons showing edema and loosening, a slight decrease in cell count, and disorderly arrangement. Cells in the CA1 zone showed significant injury, and brain parenchymal bleeding and erythrocyte leakage could be observed (Figure 5B). Given that the hippocampus is a core region for spatial memory function, the observed hippocampal neuronal damage in hypoxic rats may underlie the cognitive impairments detected in the MWM test [33]. Compared to the positive control group, the rat hippocampal tissue damage showed some improvement, the cell count significantly increased, the cell arrangement was neat, the neuronal structural abnormalities decreased, and the neuronal edema and brain parenchymal bleeding showed alleviation and improvement in the various CTE groups (Figure 5C–F). Our HE staining results demonstrated that CTE effectively attenuated hypoxia-induced hippocampal neuronal damage. This protective effect is in line with the findings of Wang et al. [21], who reported that Rhodiola crenulata aqueous extract alleviated hypoxia-induced hippocampal histopathological injury and attenuated neuronal apoptosis in a rat model of hypobaric hypoxia.

### 3.5. CTE Regulates the Hippocampal Tissue PI3K/Akt/mTOR-HIF-1α Signaling Pathway

**Figure 6:**
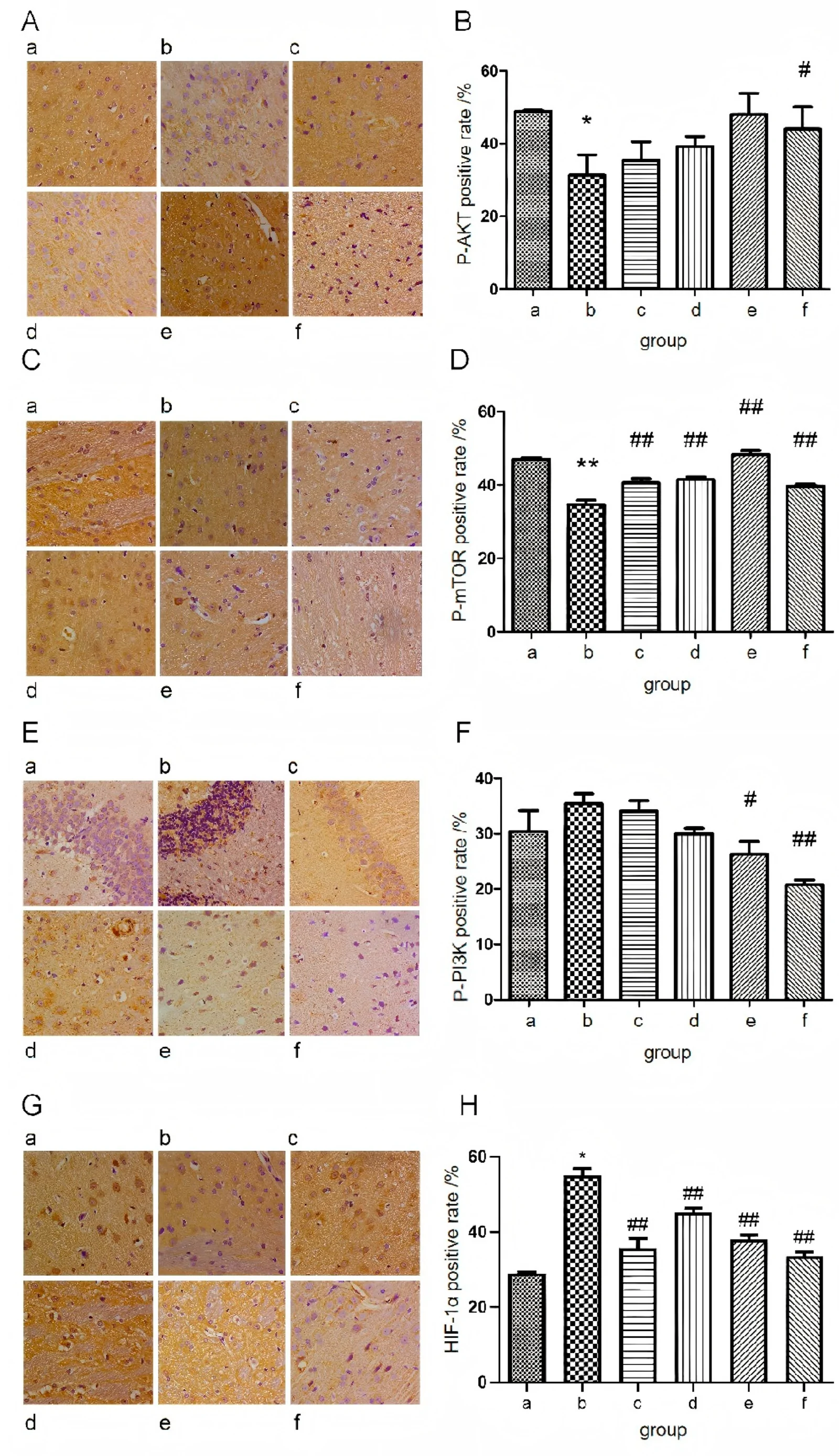
Hippocampal tissue section immunohistochemistry results (400×) obtained from different groups of rats. a: Normoxic control group; b: Model group; c: Positive control group; d: Low-dose CTE group; e: Medium-dose CTE group; f: high-dose CTE group. (**A**): P-Akt protein immunohistochemistry results. (**B**): P-Akt protein positivity rate. (**C**): P-mTOR protein immunohistochemistry results. (**D**): P-mTOR protein positivity rate. (**E**): PI3K protein immunohistochemistry results. (**F**): PI3K protein positivity rate. (**G**): HIF-1α protein immunohistochemistry results. (**H**): HIF-1α protein positivity rate. N = 10 rats per group. ** *p* < 0.01, * *p* < 0.05 compared to the normoxic control group; ## *p* < 0.01, # *p* < 0.05 compared to the model group.

The immunohistochemistry results showed that compared to the normoxic control group, the P-PI3K protein expression level increased by 5.04% and the P-Akt and P-mTOR protein expression levels decreased by 17.51% and 12.21%, respectively, after hypobaric hypoxia (Figure 6B,D,F,H). Compared to the model group, there were no significant differences in P-PI3K and P-Akt protein expression levels in the positive control group, whereas P-mTOR protein expression was significantly elevated (*p* < 0.01) (Figure 6D). Compared to the model group, the P-PI3K protein expression levels decreased by 5.47%, 9.14%, and 14.65% (Figure 6F), the P-Akt protein expression levels increased by 7.87%, 16.68%, and 12.71% (Figure 6B), the P-mTOR protein expression levels increased by 6.74%, 13.61%, and 5.00%, respectively (Figure 6D), and the HIF-1α protein expression decreased by 9.881%, 17.038% and 21.472%, respectively (Figure 6G) in the various CTE groups.

The PI3K/AKT/mTOR signaling pathway is critically involved in synaptic plasticity and memory formation, and its dysfunction has been implicated in various neurological disorders. In the present study, chronic HH exposure significantly increased the expression of P-PI3K and HIF-1α, while decreasing P-AKT and P-mTOR in the rat hippocampus, indicating a disrupted signaling axis. Although HIF-1α upregulation is an adaptive response to hypoxia, its sustained activation may exacerbate neuroinflammation and oxidative stress. Conversely, the suppression of mTOR activity is known to impair spatial cognitive function.

Administration of CTE reversed these abnormalities in a non-monotonic dose-dependent manner. The medium dose most effectively restored P-AKT and P-mTOR levels and reduced excessive HIF-1α expression, whereas the high dose showed diminished efficacy. This pattern aligns with the observed changes in oxidative stress markers (SOD, GSH, MDA) and suggests that CTE exerts neuroprotection by rebalancing the PI3K/AKT/mTOR-HIF-1α axis.

The ability of CTE to elevate P-mTOR may promote synaptic protein synthesis and dendritic spine maintenance, thereby counteracting HH-induced memory deficits. Meanwhile, the downregulation of HIF-1α likely attenuates hypoxia-driven inflammatory responses. Together with its antioxidant effects, CTE appears to orchestrate a multi-target protective response against chronic HH-induced cognitive impairment.

Consistent with the “optimal window” concept of HIF-1α regulation [34], our results show that CTE reduced the overactivated HIF-1α in chronic hypoxic rats, aligning with the differential regulatory patterns observed in Rhodiola studies where moderate HIF-1α elevation confers protection during acute hypoxia whereas its sustained overactivation drives neuropathology [21]. By restoring HIF-1α toward an optimal level, CTE effectively attenuated mitochondrial dysfunction and apoptosis, which parallels the well-documented protective effects of *R. rosea* and its active constituent salidroside in preserving mitochondrial integrity and blocking caspase-dependent cell death pathways.

The CTE-mediated HIF-1α correction, accompanied by recovery of P-AKT/P-mTOR signaling [35], likely underlies the preserved hippocampal histological integrity observed in our HE staining, consistent with Rhodiola’s known ability to suppress neuroinflammation and oxidative stress while promoting neuronal survival.

Collectively, these molecular and histological improvements translated into significant cognitive restoration in the MWM, corroborating the meta-analysis evidence that Rhodiola preparations consistently enhance learning and memory performance across diverse cognitive impairment models.

### 3.6. C. tubulosa Changes the Composition of Gut Microbiota in HH Rats

**Figure 7:**
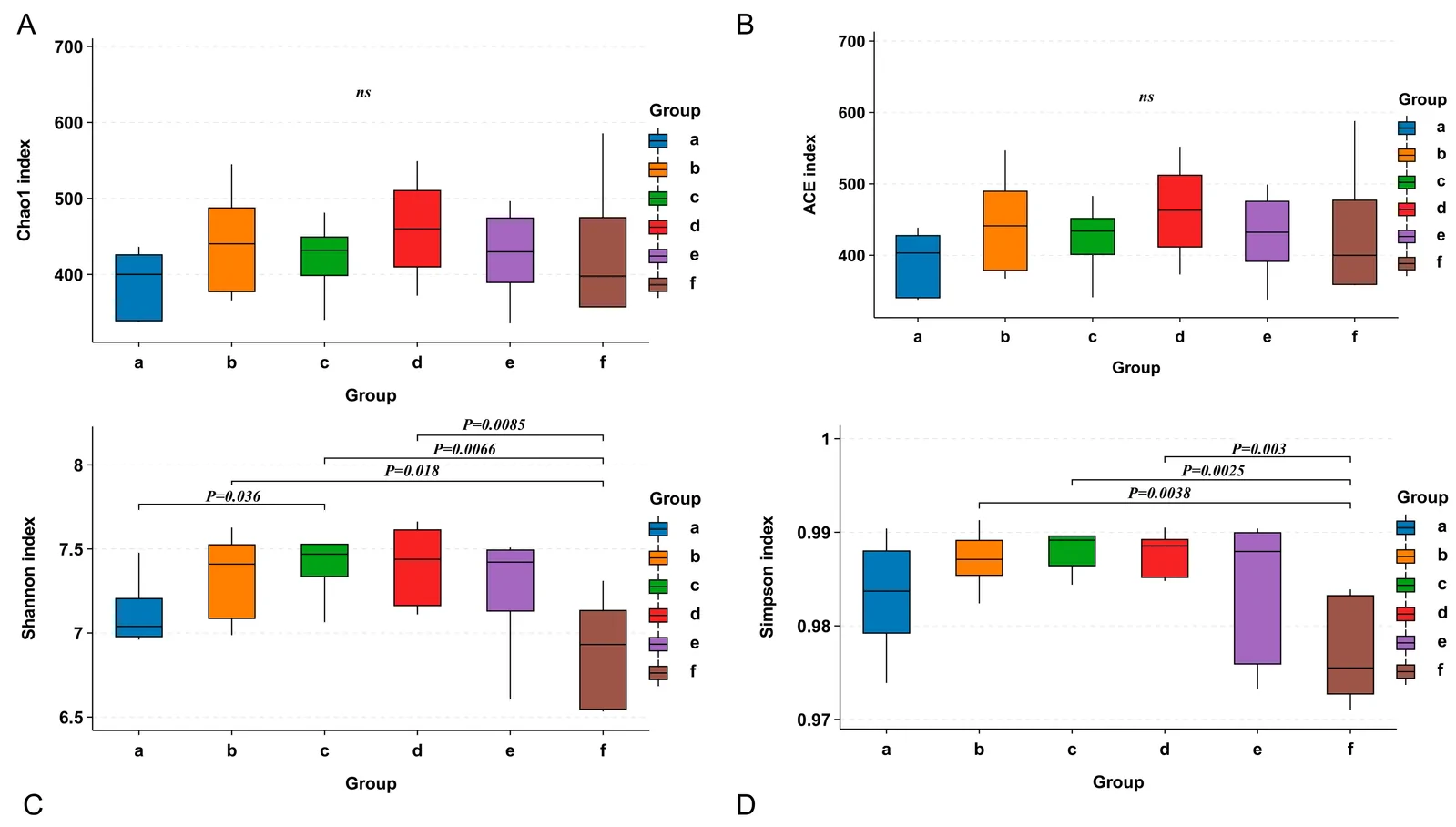
Alpha diversity analysis of different groups of rats. a: Normoxic control group; b: Model group; c: Positive control group; d: Low-dose CTE group; e: Medium-dose CTE group; f: high-dose CTE group (*n* = 6). (**A**): Chao1 index. (**B**): ACE index. (**C**): Shannon index. (**D**): Simpson index. Data are presented as mean ± SD. ns, not significant.

To evaluate the effects of CTE on gut microbial community structure, alpha diversity indices were analyzed using 16S rRNA sequencing (Figure 7). The ACE and Chao1 indices, reflecting species richness, were slightly increased under hypobaric hypoxia. In contrast, the medium- and high-dose CTE groups showed significantly decreased ACE and Chao1 indices, in some cases returning to normoxic levels. Similarly, the Shannon and Simpson indices, reflecting species diversity, were increased by hypobaric hypoxia, whereas high-dose CTE significantly reduced them. These results indicate that CTE, particularly at high doses, counteracts the abnormal elevation of gut microbial richness and diversity induced by chronic HH exposure.

### 3.7. Beta Diversity Analysis

**Figure 8:**
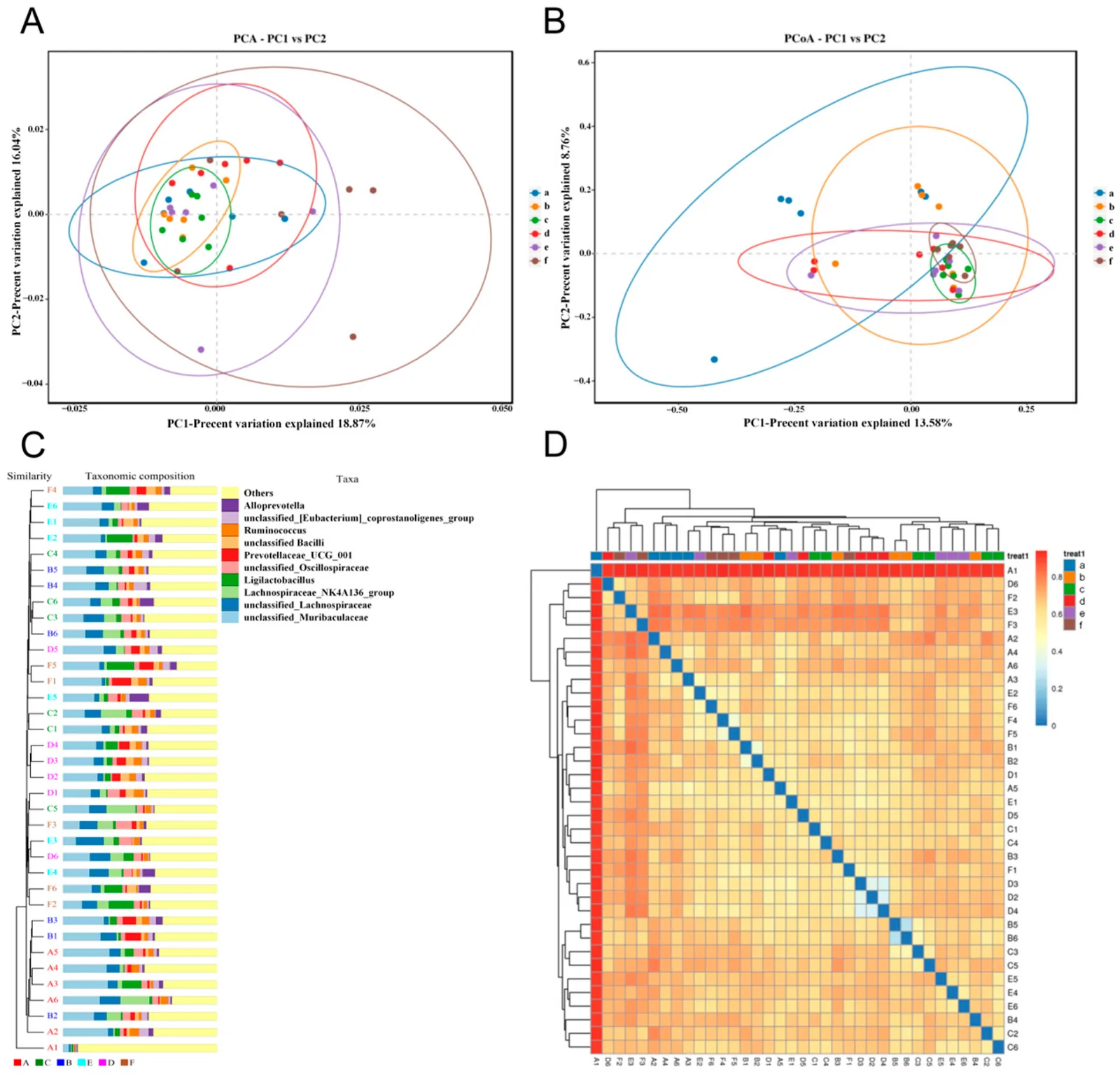
Beta diversity analysis of different groups of rats. a: Normoxic control group; b: Model group; c: Positive control group; d: Low-dose CTE group; e: Medium-dose CTE group; f: high-dose CTE group (*n* = 6). (**A**): PCA graph. (**B**): PCoA graph. (**C**): UPGMA cluster tree histogram graph. (**D**): Sample clustering heatmap.

Principal component analysis (PCA) showed that medium- and high-dose CTE treatments changed the composition of gut microbiota in HH rats (Figure 8A). In the PCoA graph, the distances between the hypoxia model group and the other groups were large, indicating that there were differences in gut microbiota composition between the hypoxia model group and the other groups (Figure 8B). To further study the effects of CTE on gut microbiota composition, we calculated the abundance at the species level in various groups using a histogram (Figure 8C) to determine the similarity in species abundance between samples. The results showed that some changes occurred in the gut microbiota structure between the normoxic control group and the model group, and there were some differences in species diversity. Additionally, there were large changes in microbiota structure in the medium- and high-dose CTE groups, and the species diversity and abundance similarity also showed large differences. Ligilactobacillus was significantly increased in the high-dose CTE group, where it helped to supplement beneficial bacteria in the gut and suppress the colonization and proliferation of harmful bacteria. Unclassified Oscillospiraceae was also significantly increased and could maintain gut health and improve immunity. In summary, high-dose CTE can intervene in the gut microbiota composition of HH rats and increase the synthesis of beneficial bacteria in their guts.

Heatmap analysis showed that the distances between the hypoxia model group and the low- and medium-dose CTE groups were longer than those between the other groups, and the intergroup sample distances were closed, demonstrating that low- and medium-dose CTE has greater effects on HH rats (Figure 8D).

### 3.8. Gut Microbiota Species Composition Analysis

**Figure 9:**
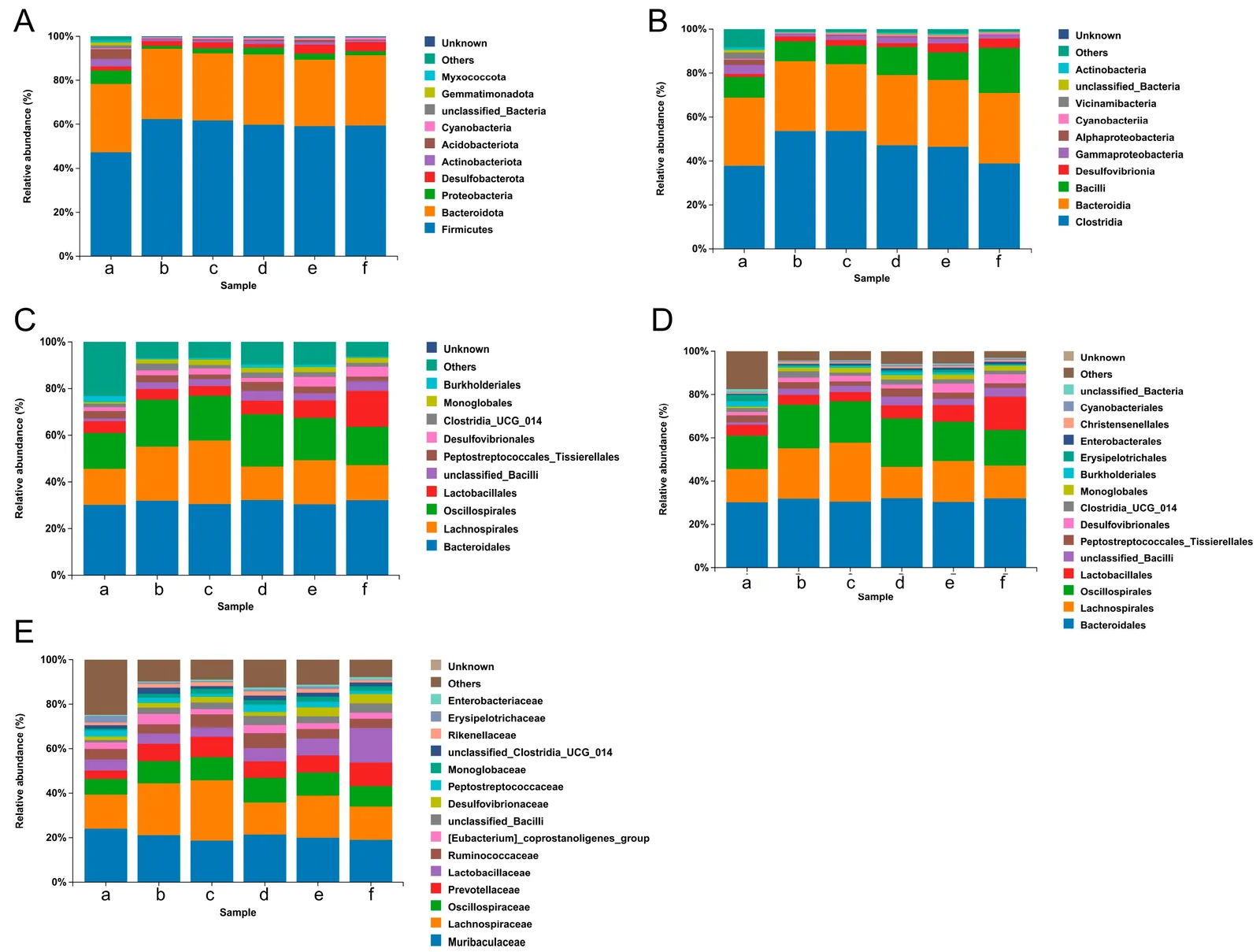
Gut microbiota species composition analysis of different groups of rats. a: Normoxic control group; b: Model group; c: Positive control group; d: Low-dose CTE group; e: Medium-dose CTE group; f: high-dose CTE group (*n* = 6). (**A**): Gut microbiota analysis at the phylum level. (**B**): Gut microbiota analysis at the class level. (**C**): Gut microbiota analysis at the order level. (**D**): Gut microbiota analysis at the family level. (**E**): Gut microbiota analysis at the genus level.

To further study the changes in gut microbiota composition in different groups, we calculated the relative abundance of gut microbiota at the phylum, class, order, family, and genus levels. After treatment with CTE, the relative levels of beneficial bacteria, such as *Bacteroidetes*, *Actinobacteria*, and *Acidobacteriota*, increased, whereas those of harmful bacteria, such as *Firmicutes*, decreased (Figure 9A). At the class level, CTE treatment decreased the level of Clostridia and increased the levels of *Bacilli* and *Bacteroidia* (Figure 9B). At the order level, CTE treatment increased the level of beneficial bacteria *Lactobacillales* and decreased the level of harmful bacteria *Clostridiales* (Figure 9C). At the family level, CTE treatment increased the levels of beneficial bacteria *Muribaculaceae*, *Oscillospiraceae*, and *Acidobacteriaceae*, and decreased the levels of harmful bacteria *Lachnospiraceae* (Figure 9D). At the genus level, CTE treatment increased the levels of beneficial bacteria *Muribaculaceae* and *Oscillospira*, and decreased the levels of harmful bacteria *Lachnospira* (Figure 9E). This demonstrates that CTE can increase the ratio of beneficial to harmful bacteria in the gut. Our results revealed that CTE administration was associated with compositional changes in the gut microbiota of hypoxic rats.

## 4. Discussion

A reliable animal model is essential for studying high-altitude hypoxia-induced cognitive impairment and evaluating therapeutic interventions. Here, we established a hypobaric hypoxia model in rats using a hypobaric hypoxia chamber, a protocol consistent with established paradigms [11,34,35,36]. Model validity was confirmed by significant MWM deficits and hippocampal CA1 neuropathology. *R. rosea* extract was included as a positive control to benchmark CTE efficacy. By adopting a hypoxia protocol parallel to that in prior Rhodiola studies, we ensured comparable conditions, enabling direct comparison of CTE’s therapeutic effects with this reference standard [16].

The MWM confirmed that chronic hypobaric hypoxia significantly impaired spatial learning and memory [36]. CTE administration reversed these deficits in a non-monotonic dose-dependent manner, with the medium dose yielding the most pronounced improvement-escape latency reduced by 61.53%, center activity time increased by 101.52%, and platform crossings increased by 300.00%. CTE also enhanced cognitive performance under normoxic baseline conditions, suggesting both corrective and fundamental cognition-enhancing properties. These behavioral improvements are comparable to *R. rosea* and salidroside, which significantly shortened escape latency and increased target quadrant exploration in a meta-analysis of 28 studies.

Immunohistochemistry revealed that chronic hypoxia significantly increased HIF-1α expression in the hippocampus, while CTE treatment effectively reduced overactivated HIF-1α to a more optimal level, consistent with the differential HIF-1α modulation observed in Rhodiola studies-promoting HIF-1α accumulation in acute hypoxia [21] versus reversing excessive accumulation in chronic hypoxia. Concomitantly, hypoxia suppressed PI3K, AKT, and mTOR phosphorylation, indicating inactivation of pro-survival signaling [37]. CTE significantly restored their phosphorylation, reinstating survival signaling and promoting protein synthesis necessary for synaptic remodeling. This is consistent with mTORC1/2 involvement in spatial cognitive function [34,36]. The coordinated regulation of HIF-1α and mTOR by CTE likely re-establishes the homeostatic equilibrium required for neuronal survival.

HE staining revealed that chronic hypoxia-induced marked histopathological alterations in hippocampal CA1-neuronal disarray, edema, reduced cell count, and hemorrhage [38]. CTE administration significantly attenuated these changes, with the medium dose showing the most pronounced protection. This structural preservation is attributable to restored AKT/mTOR survival signaling and attenuated oxidative stress, as evidenced by our immunohistochemical and biochemical data. Similar hippocampal neuroprotection has been reported for *R. rosea* and salidroside, which attenuated hypoxia-induced neuronal apoptosis and preserved hippocampal architecture [18,21]. The consistency between histopathology and behavior strongly supports that CTE preserves cognitive function through hippocampal integrity protection.

Oxidative stress is a hallmark of high-altitude hypoxia-induced brain injury [2,32,39]. Chronic hypoxia significantly decreased SOD and GSH-Px activities while increasing MDA, indicating depletion of endogenous antioxidant defenses and enhanced lipid peroxidation. CTE treatment effectively reversed these changes, consistent with the documented free radical-scavenging properties of salidroside [6]. This antioxidant effect likely contributes to the preservation of neuronal membrane integrity observed in HE staining, and together with the immunochemical data, supports a model in which CTE attenuates oxidative damage and complements the restored survival signaling to protect hippocampal neurons.

Emerging evidence highlights the gut microbiota as a key modulator of brain function through the microbiota–gut–brain axis under hypoxic conditions. Hypobaric hypoxia exposure significantly alters gut microbial composition, and fecal microbiota transplantation from hypoxic mice replicates memory deficits in recipients [40]. Probiotic interventions have been shown to alleviate hypoxia-induced cognitive impairment by restoring gut barrier integrity and reducing systemic inflammation. As the CTE concentration increased, the number of Lactobacillus also gradually increased, and the lactobacillus-derived EV could alter BDNF expression in the hippocampus. Therefore, we hypothesized that CTE may affect the hippocampal tissue and improve memory by regulating the proportion of beneficial bacteria in the gut microbe, as well as through the derived products of beneficial bacteria.

In summary, our findings establish a multi-level framework for CTE-mediated neuroprotection against chronic hypobaric hypoxia-induced cognitive impairment alongside potential gut microbiota modulation. The consistency of our results with the well-documented neuroprotective profile of *R. rosea* supports CTE as a promising candidate for preventing high-altitude cognitive dysfunction.

### Limitations

Although pooled MWM escape latency data failed to detect significant gender differences after excluding mixed-sex trials, rodents exhibit evident sex-specific cognitive divergence, with males predominating in spatial working memory while females excel in visual and social cognition. Mixed-sex cohorts may increase experimental heterogeneity. For the sake of minimizing confounding variables and guaranteeing consistent experimental results, only male rats were adopted herein. Nevertheless, this single-sex experimental design constitutes an obvious limitation of the current work. We cannot determine whether the neuroprotective efficacy of CTE against high-altitude hypoxic cognitive impairment differs between male and female rats, nor can we identify sex-specific discrepancies in hippocampal oxidative stress and gut microbiota remodeling induced by chronic hypoxia. In follow-up relevant research, both male and female rodent models should be incorporated to systematically compare sex-dependent pharmacological responses to CTE and fully clarify its anti-hypoxia mechanisms. Additionally, this study only simulated static high-altitude hypoxic conditions without combining cold stress and high-intensity exercise, which are common in real highland environments; combined stress models may alter the magnitude of CTE’s regulatory efficacy on the PI3K/Akt/mTOR-HIF-1α pathway and gut–brain axis homeostasis.

For human translation, hypoxia-triggered brain and intestinal lesions in rats mirror clinical symptoms of long-term highland residents. *R. rosea* is the conventional first-line herbal agent for altitude sickness prevention in high-altitude crowds, yet resource scarcity limits its extensive use. Notably, CTE produced equivalent protective effects against hypoxic cognitive impairment as *R. rosea* in this study, revealing strong substitution potential of *C. tubulosa* as an alternative anti-hypoxia natural supplement. Its bioactive ingredients act on evolutionarily conserved mammalian neuroprotective pathways. Still, interspecies differences in metabolism and effective doses prevent direct dose translation from rats to humans. Only young male rats were tested here; further animal and clinical research is needed to confirm whether these protective effects translate to females, the elderly and people with chronic cardio-cerebrovascular disorders.

## 5. Conclusions

In summary, we established a HH rat model and treated the animals with CTE. Our results showed that CTE treatment increased SOD and GSH activities and decreased MDA levels in both brain tissue and serum. Additionally, CTE decreased the expression of P-PI3K protein while increasing the expression of P-Akt and P-mTOR proteins in the brain. These findings suggest that CTE protects hippocampal tissue in HH rats, possibly through the PI3K/Akt/mTOR-HIF-1α signaling pathway. Furthermore, high-throughput sequencing was employed to analyze the effects of CTE on the gut microbiota of HH rats. Gut microbiota composition was examined at five taxonomic levels (phylum, class, order, family, and genus). The results demonstrated that CTE increased the ratio of beneficial bacteria and decreased the ratio of harmful bacteria in the gut. Collectively, CTE ameliorated memory decline induced by hypobaric hypoxia in rats, exerted protective effects against cognitive impairment, and modulated gut microbiota composition.

## Figures and Tables

**Table 1:** Effects of *C. tubulosa* extract (CTE) on hippocampal tissue and serum SOD activity (x ± s, *n* = 10).

Group	Hippocampus (nmol/mg prot)	Serum (μmol/L)
Normoxic control group	390.15 ± 29.30	472.02 ± 10.65
Model group	288.48 ± 45.62 **	378.84 ± 18.12 **
Positive control group	357.88 ± 29.41 ##	411.78 ± 17.52 ##
Low dose CTE	322.84 ± 28.53	406.83 ± 20.58 #
Medium dose CTE	348.99 ± 20.22 ##	413.07 ± 28.19 ##
High dose CTE	357.42 ± 40.98 ##	391.43 ± 20.72

Note: ** *p* < 0.01 compared to the normoxic control group; ## *p* < 0.01, # *p* < 0.05 compared to the model group.

**Table 2:** Effects of CTE on hippocampal tissue and serum GSH-PX activity (x ± s, *n* = 10).

Group	Hippocampus (nmol/mg prot)	Serum (μmol/L)
Normoxic control group	35.26 ± 2.48	11.27 ± 0.80
Model group	22.86 ± 2.12 **	8.01 ± 0.57 **
Positive control group	28.99 ± 2.85 ##	9.40 ± 0.80 ##
Low dose CTE	27.86 ± 2.72 ##	8.48 ± 0.67
Medium dose CTE	31.16 ± 2.60 ##	10.08 ± 0.64 ##
High dose CTE	28.35 ± 2.14 ##	10.11 ± 1.12 ##

Note: ** *p* < 0.01 compared to the normoxic control group; ## *p* < 0.01, compared to the model group.

**Table 3:** Effects of CTE on hippocampal tissue and serum GSH-PX MDA levels (μmol/L) (x ± s, *n* = 10).

Group	Hippocampus (nmol/mg prot)	Serum (μmol/L)
Normoxic control group	3.51 ± 0.51	7.66 ± 0.84
Model group	5.68 ± 0.90 **	11.86 ± 1.23 **
Positive control group	4.24 ± 0.57 ##	9.28 ± 1.09 ##
Low dose CTE	4.68 ± 0.67 #	9.87 ± 0.37 ##
Medium dose CTE	3.28 ± 0.89 ##	9.29 ± 1.06 ##
High dose CTE	3.72 ± 0.66 ##	10.86 ± 0.53

Note: ** *p* < 0.01 compared to the normoxic control group; ## *p* < 0.01, # *p* < 0.05 compared to the model group.

## Data Availability

The raw sequencing data generated in this study have been deposited in the NCBI Sequence Read Archive (SRA) under accession number PRJNA1481623. The data are publicly available and can be accessed via the following link: https://www.ncbi.nlm.nih.gov/bioproject/?term=PRJNA1481623 (accessed on 24 June 2026). The original contributions presented in this study are included in the article. Further inquiries can be directed to the corresponding author.

## Abbreviations

The following abbreviations are used in this manuscript:

HH	Hypobaric hypoxia
CTE	*Cistanche tubulosa* extracts
CPhGs	*Cistanche* Phenylethanol glycosides
MWM	Morris water maze
SOD	Superoxide dismutase
GSH-Px	Glutathione peroxidase
MDA	Malondialdehyde
ANOVA	Analysis of variance
Ech-Gs	Echinacoside glycosides
Act-Gs	Acteoside glycosides
PCA	principal component analysis
PCoA	principal coordinate analysis
